# Enhanced Capacitive Performance of N-Doped Activated Carbon from Petroleum Coke by Combining Ammoxidation with KOH Activation

**DOI:** 10.1186/s11671-016-1460-3

**Published:** 2016-05-11

**Authors:** Yan Zhang, Yu Zhang, Jufeng Huang, Dongfeng Du, Wei Xing, Zifeng Yan

**Affiliations:** State Key Laboratory of Heavy Oil Processing, Key Laboratory of Catalysis, CNPC, School of Chemical Engineering, China University of Petroleum, Qingdao, 266580 People’s Republic of China; School of Science, State Key Laboratory of Heavy Oil Processing, China University of Petroleum, Qingdao, 266580 People’s Republic of China

**Keywords:** Petroleum coke, Activated carbon, Nitrogen doping, Supercapacitor

## Abstract

Low cost with high specific capacitance and energy density is the critical and main requirement for practical supercapacitors. A novel N-doped activated carbon was fabricated by KOH activation of petroleum coke and ammonia treatment. The as-prepared carbon exhibits a high specific surface area (1875 m^2^ g^−1^), excellent conductivity (57 S m^−1^), and rich nitrogen level (4.0 wt%). Those outstanding characters result in this porous carbon a hopeful electrode material for electrochemical supercapacitors. It shows high specific capacitance (up to 299 F g^−1^) and superior rate capability (76 % retention ratio at 20 A g^−1^) in 30 wt% KOH aqueous electrolyte. This efficient treatment method ensures its prosperous application in energy storage systems.

## Background

The growing worldwide energy consumption and consequent raising in concerns on environmental issues derived from excessive reliance on fossil fuels are prompting to develop renewable energy sources, e.g., solar and wind energy systems. However, due to the fact that these energies are heavily fluctuating, reliable and cost-efficient conversion and storage of electricity devices have to be developed to realize uninterrupted and balanced electric energy supply [1, 2]. Supercapacitors have been considered to be promising for energy storage and conversation because of outstanding power density, excellent cycle stability, and low cost [3]. The electrode materials exert a key role in the supercapacitor performance. Recently, tremendous work has been carried out to research different carbons as electrode materials, and among these carbon materials, activated carbon (AC) is the most commonly used electrode material because of its high specific surface area, superior electrical conductivity, and cost effectiveness [4].

To date, the utilization of mineral (asphalt, petroleum coke, and coal) as an AC precursor has become increasingly valuable for its rich storage, low cost, and high carbon content [5, 6]. A high additional value can be acquired with further use of AC. Chemical activation using alkali (NaOH and KOH) has been widely applied for the preparation of AC, which can result in carbons with high specific surface areas and electrochemical double layer (EDL) capacitance [7–10]. However, for the conventional AC, specific capacitance and energy density are rather limited because the energy storage mechanism is onefold EDL capacitance.

In order to solve this problem, the pseudocapacitive properties can be introduced by creating surface functional groups [11]. For example, treatments at elevated temperatures by mixtures of gases containing ammonia (Ar-NH_3_, N_2_-NH_3_, etc.) have been proposed to modify the surface functionality and porous texture [12–14]. This will allow these materials to acquire useful redox properties that can further promote the total capacitance through pseudocapacitance effect [15]. At the same time, surface functional groups could enhance the wettability and expand the accessible surface area of electrode materials [16]. It is found that nitrogen doping can be a promising strategy to enhance capacitance while maintaining superb cycle endurance [17]. Besides, doped nitrogen could promote the conductivity of carbon materials, which benefits rate property and power density of carbon-based supercapacitors [18].

In this work, N-doped activated carbon (NOAC) has been synthesized by KOH activation of petroleum coke (PC), followed by ammonia treatment in order to improve the capacitive properties and thus enhance their energy storage performance as electrode materials. Effects of pre-oxidization (HNO_3_) on the surface chemistry of AC were also studied. The result shows that ammonia treatment temperature could affect the composition and density of the formed surface functional groups and also porous texture of the as-prepared materials. The introduced functional groups not only enhance the EDL capacitance but also provide pseudocapacitance, leading to an enhanced overall capacitance.

## Methods

### Materials

A typical Chinese PC from Shengli Refinery was used as the carbon precursor. The parent material was ground and sieved to choose the particle size within the range of 100–150 μm. The obtained powder was used as the precursor of AC after drying in an oven at 100 °C for 12 h. KOH, HNO_3_, and HCl were purchased from Sinopharm Chemical Reagent Co., Ltd., and used without purification.

### Synthesis of AC Derived from PC

In a typical KOH activation process, 10 g of PC was firstly mixed with KOH at a KOH/PC mass ratio of 3:1. The mixture obtained was then transferred into a crucible and activated at 800 °C for 2 h with a heating ramp of 5 °C min^−1^ in argon flow before cooling down to room temperature. The obtained sample was washed with distilled water until the pH of the filtrate was ~7. The final products were dried overnight at 80 °C and denoted as ACs.

### Pre-oxidization of AC

Five grams of AC powder was pre-oxidized in 50 mL 20 wt% HNO_3_ under continuous stirring for 3 h at 60 °C and washed with deionized water until the pH was neutral. The products were dried overnight at 80 °C and named as OAC.

### Chemical Modification by Ammonia

For modification by ammonia, the treatment was carried out at fixed temperatures from 400 to 800 °C for 2 h. For simplicity, the nitrogen-doped porous carbons were denoted as NOACX, where X denotes the activation temperature. The as-prepared samples were denoted as NOAC400, NOAC500, NOAC600, NOAC700, and NOAC800. For example, sample NOAC400 was prepared as follows: 2 g OAC was placed in a porcelain boat inside a horizontal pipe reactor. During the heating process, Ar flow was firstly used with a heating rate of 5 °C min^−1^. Ar was switched into NH_3_ when the temperature reaches 400 °C, and the activation process is maintained at 400 °C for 2 h. After activation, the solid samples were cooled down to room temperature under the Ar flow. After the KOH activation of PC and ammonia treatment, the total carbon yield is about 84 %.

### Material Characterization

Morphologies of the carbon samples were observed by field emission scanning electron microscopy (SEM, FEI Sirion 200, Netherlands) and transmission electron microscopy (TEM, JEOL-2100UHR, Japan). The surface chemical properties were characterized by Fourier transform infrared (FT-IR) spectroscopy (Nicolet 6700, Thermo Scientific) with potassium bromide pellets and X-ray photoelectron spectroscopy (XPS, PHI 5000 VersaProbe, ULVAC-PHI, Japan) with an Al Kα X-ray source (1486.6 eV). An elemental analyzer (ANTEK, ANTEK 9000, USA) was used to investigate the elemental content of the synthesized samples. Nitrogen adsorption-desorption measurements were performed on a Tristar 3000 analyzer (Micromeritics, USA) to acquire specific surface area and pore-structure parameters. Employing the multipoint Brunauer-Emmett-Teller (BET) method, the specific surface area (*S*_BET_) was calculated at *P*/*P*_0_ values between 0.06 and 0.3. The total micropore volume (*V*_micro_) was calculated from the N_2_ adsorption data using the *t*-plot method, and the total pore volume (*V*_total_) was evaluated from the amount of liquid nitrogen adsorbed at *P*/*P*_0_ of 0.99. The pore size distributions (PSD) were estimated via the density functional theory (DFT) using N_2_ adsorption data. To avoid diffusion problems of N_2_ molecules at 77 K (−196 °C) inside the narrow micropores, CO_2_ adsorption isotherms were tested by a volumetric determination method at 273 K (0 °C) using an ASAP 2010 physisorption analyzer (Micromeritics, USA) to evaluate narrow microporosity (pore size <1 nm).

### Electrochemical Measurement

Electrochemical performance of NOAC for supercapacitors was examined with cyclic voltammetry (CV), galvanostatic charge/discharge (GCD), and electrochemical impedance spectroscopy (EIS) measurement. All the data were collected with a three-electrode system using a CHI660D testing station. The working electrode was made up of active material (NOAC) and polytetrafluoroethylene (PTFE) at a weight ratio of 95:5. The mixture was dispersed via sonication for 15 min and dried overnight at 80 °C. Then, a nickel foam was coated with the mixture with an area of 1 cm^2^ and pressed at 10 MPa to form a slice as a working electrode. The loading amount for each electrode was ~2 mg. While the platinum film was adapted as the counter electrode, a saturated calomel electrode (SCE) was used as the reference electrode. A solution of 30 wt% KOH aqueous electrolyte was employed. The CV and GCD tests were measured at a potential region from −1 to 0 V (vs. SCE). The specific capacitance (*C*) was calculated on the basis of the discharge curve data according to the equation below:

$$ C=\frac{I\varDelta t}{m\varDelta V} $$

where *I* is the discharge current (A), *Δt* is the discharge time (s), *m* is the mass loading of an active material in one electrode (g), and *ΔV* is the potential window (V).

## Results and Discussion

**Fig. 1:**
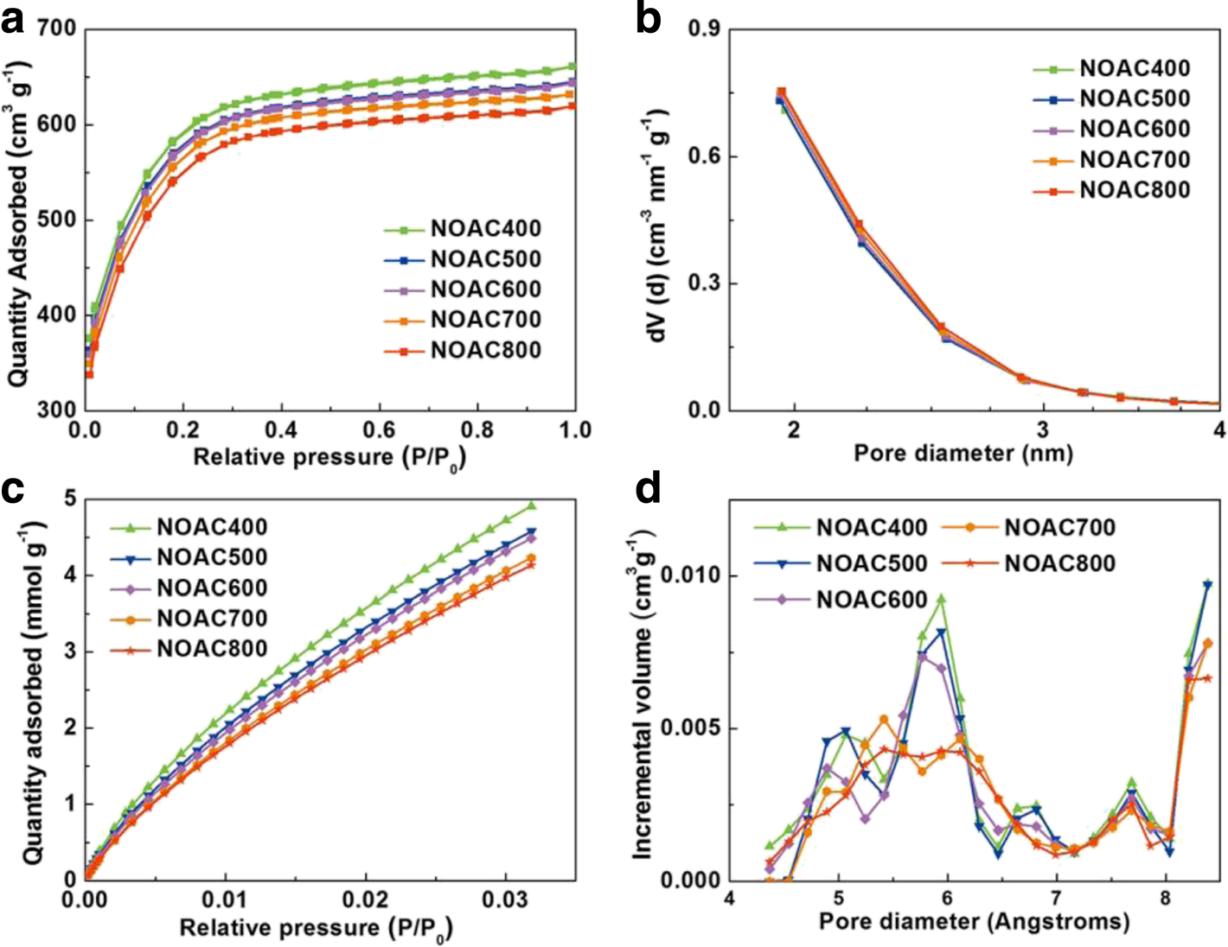
**a** Nitrogen adsorption/desorption isotherms measured at 77 K and **b** pore size distributions of NOAC at different temperatures. **c** Low-pressure CO_2_ physisorption isotherms measured at 273 K for the carbons and **d** NLDFT CO_2_ micropore size distributions

The BET specific surface area and pore-structure parameters of the as-prepared carbons were calculated by N_2_ adsorption-desorption analysis. N_2_ adsorption-desorption isotherms (Fig. 1a) of all the NOAC samples are typical type I (IUPAC), indicating microporous nature. It is obvious that for NOAC, the adsorption capacity decreases slowly with the increase of activation temperature, reflecting the decrease of the pore volume. The pore-structure parameters of these carbons are exhibited in Table 1. The BET specific surface area of NOAC400, NOAC500, NOAC600, NOAC700, and NOAC800 are 1875, 1836, 1837, 1817, and 1780 m^2^ g^−1^, respectively. Sample NOAC400 possesses the highest specific surface area and pore volume compared with the other carbons prepared at higher temperatures. The high surface area could provide a large adsorption interface for electrolyte ions to form the EDL, which is beneficial for its supercapacitor application.

**Table 1:** Surface area and pore-structure parameters of the as-prepared samples

Samples	*S*_BET_^a^	*S*_Micro_^b^	*S*_Meso_^c^	*V*_total_^d^	*V*_Micro_^e^	*V*_Meso_^f^	APD^g^
	m^2^ g^−1^	m^2^ g^−1^	m^2^ g^−1^	cm^3^ g^−1^	cm^3^ g^−1^	cm^3^ g^−1^	nm
NOAC400	1875	660	737	1.02	0.38	0.45	2.18
NOAC500	1836	594	726	1.00	0.35	0.44	2.17
NOAC600	1837	568	795	1.00	0.33	0.47	2.17
NOAC700	1817	521	763	0.98	0.30	0.46	2.15
NOAC800	1780	465	768	0.96	0.27	0.46	2.15

^a^BET surface areas

^b^Micropore surface areas calculated by *t*-plot method

^c^Mesopore surface areas equal to *S*_BET_ minus *S*_Micro_

^d^Total pore volume of pores at *P*/*P*_0_ = 0.99

^e^The *t*-plot micropore volume

^f^The BJH adsorption cumulative volume of pores

^g^The average pore size calculated by 4*V*_Total_/*S*_BET_

A CO_2_ physisorption test was conducted to get better understanding of the pore size distribution of less than 1 nm. Substantial microporosity is visible when CO_2_ is used as an adsorbate (Fig. 1c, d). The decrease of adsorbing capacity and pore volume is observed when the OAC is treated by ammonia at higher temperatures. This may be due to the fact that high-temperature ammonia treatment leads to the collapse of ultra-small micropores, which is negative for its electrochemical capacitive performance [19].

**Fig. 2:**
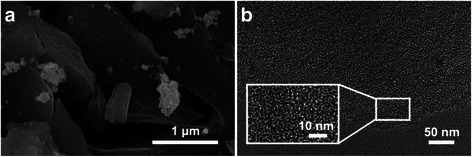
SEM (**a**) and TEM (**b**) images of NOAC400. Inset is a high-resolution TEM image

Figure 2 illustrates microscopic morphology of NOAC400. From the SEM image (Fig. 2a), the sample exhibits a blocky shape, which originates from the grinding process of raw PC. As shown in the TEM image (Fig. 2b), the abundant micropores of NOAC400 can be observed, which is derived from KOH activation at a high temperature. This result agrees well with the N_2_ adsorption data. A high-resolution TEM image (inset of Fig. 2b) confirms the existence of short graphene layers with partially ordered arrangement, and this kind of structure is helpful to enhance the conductivity of NOAC400.

**Fig. 3:**
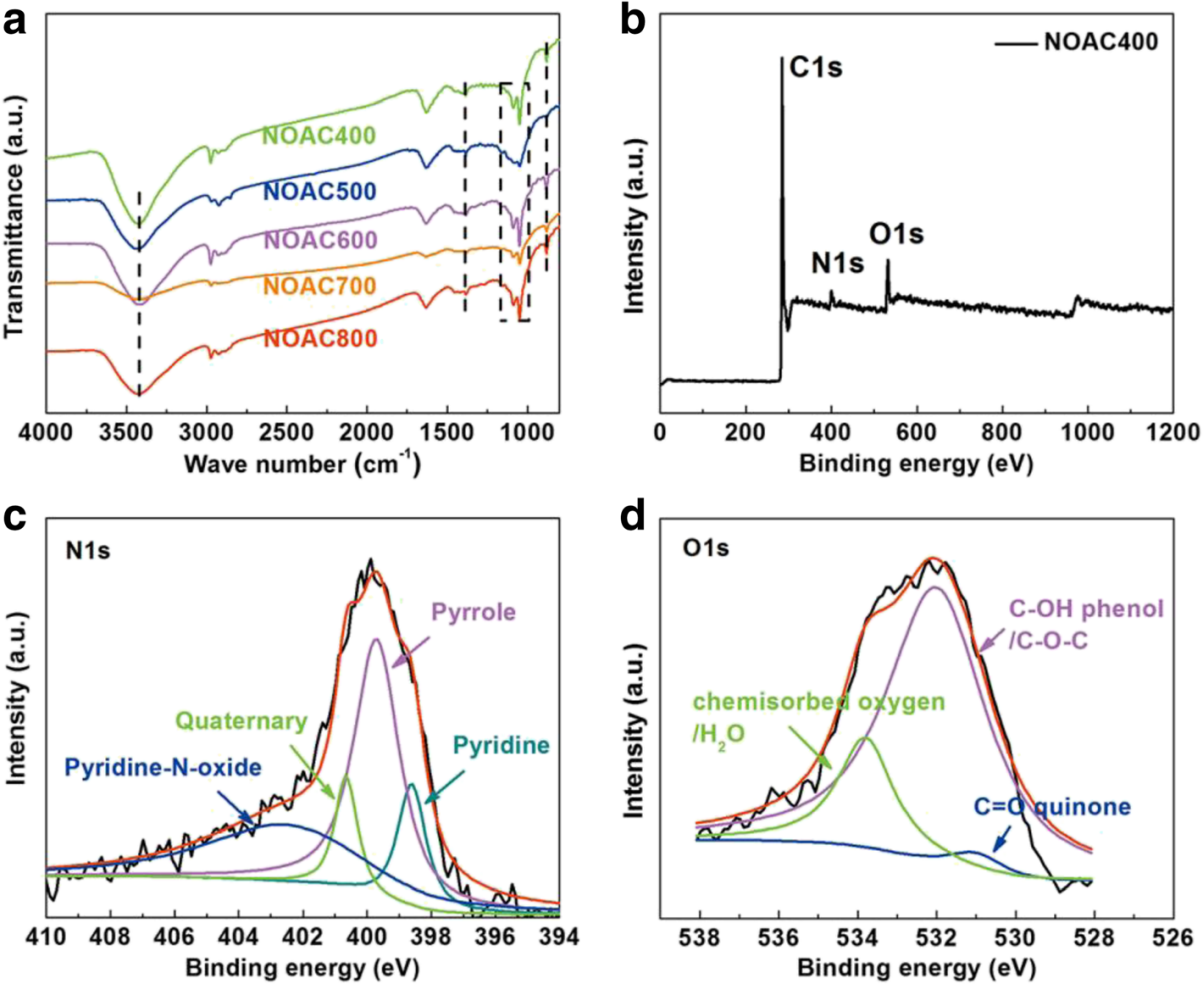
**a** FT-IR spectra of NOAC at different temperatures. **b** XPS survey spectra of NOAC400. High-resolution XPS spectra of N 1s peak (**c**) and O 1s peak (**d**) for NOAC400

The FT-IR spectra (Fig. 3a) of the samples were recorded to verify the successful introduction of surface functional groups. The broad bands at about 3433 cm^−1^ were associated with the –O–H and –N–H stretching vibration modes. Peaks at 1387 cm^−1^ can be attributed to symmetrical stretching vibration of –NO_2_. In addition, 1048 and 881 cm^−1^ are related to –NH_2_ vibration bands. All these characters indicate the existence of surface N-containing and O-containing functional groups.

Elemental analysis (EA) and X-ray photoelectron spectroscopy were conducted to investigate the chemical composition. From the results of the EA analysis, the nitrogen content of NOAC400 is 4 wt% (Table 2), which is the highest compared with the other samples. The chemical bonding nature of nitrogen and oxygen species was examined with X-ray photoelectron spectroscopy (XPS). The N 1s XPS spectrum of NOAC is illustrated in Fig. 3c, which could be deconvoluted to four types of nitrogen species: pyridinic (398.7 eV), pyrrolic (400.3 eV), quaternary (401.4 eV), and pyridine-N-oxide (402.8 eV) nitrogen, respectively. Among these four types of nitrogen, pyrrolic-type and pyridinic-type nitrogen account for 78 at% of the entire nitrogen content, which could be beneficial to promote pseudocapacitance effect [20]. As shown in Fig. 3d, NOAC400 also possesses high content of oxygen species, mainly deriving from the KOH activation process. The binding energy around 531, 532, and 533.8 eV represents C=O quinone type groups, C–OH phenol groups and/or C–O–C ether groups, and chemisorbed oxygen (COOH carboxylic groups) and/or water, respectively [13]. Abundant N and O functional groups can introduce extra pseudocapacitance and facilitate the wettability of carbon surface in aqueous electrolytes.

**Table 2:** Elemental composition evaluated from EA and conductivity of the as-prepared samples

Samples	C%	N%	O%	C/N	Conductivity
					S m^−1^
NOAC400	89.4	4.0	5.7	22.4	57
NOAC500	91.4	3.4	4.2	26.9	48
NOAC600	93.4	3.2	2.6	29.2	46
NOAC700	94.3	2.9	2.1	32.5	52
NOAC800	95.0	2.1	2.0	45.2	51

### Electrochemical Capacitive Performance

**Fig. 4:**
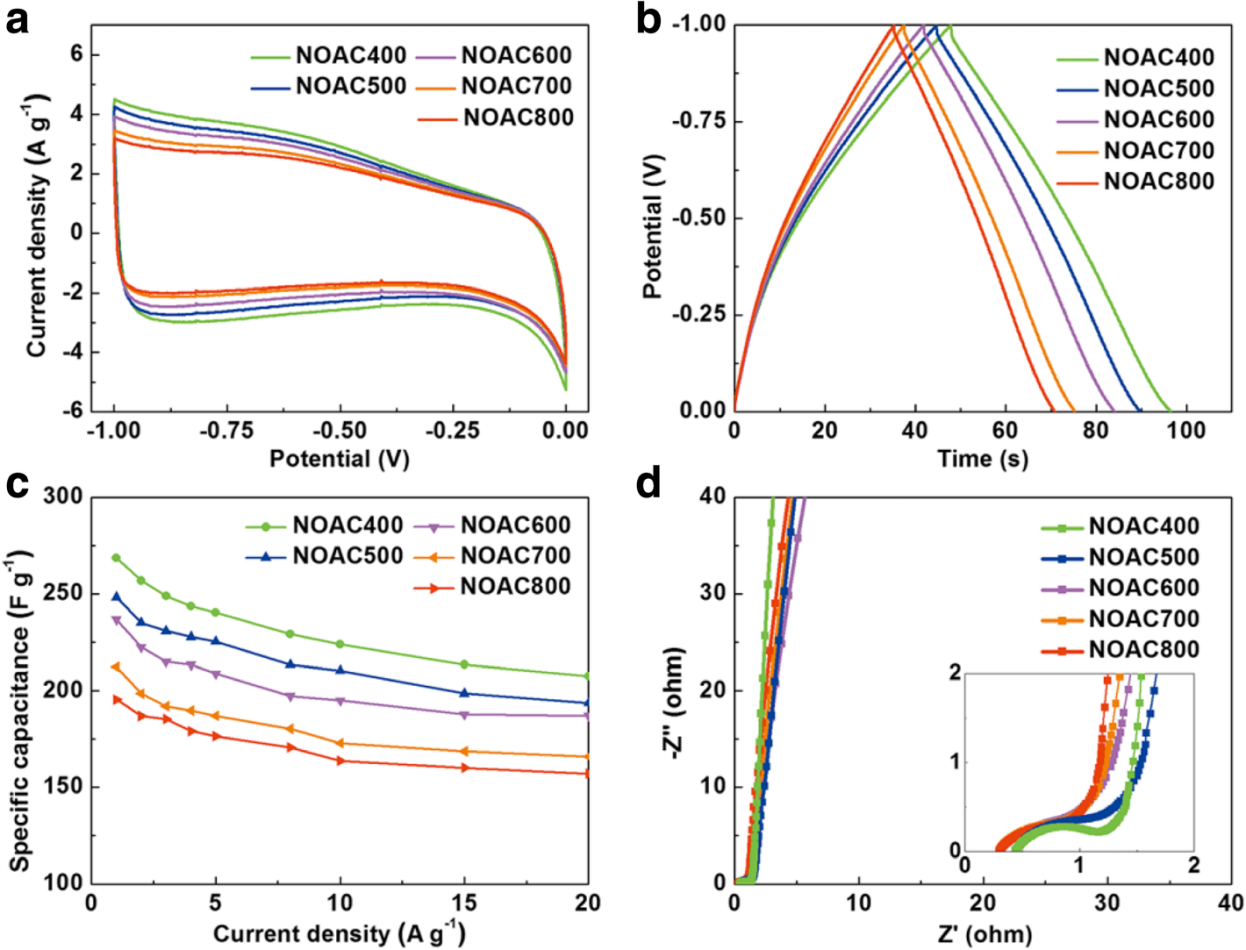
**a** CV curves at a scan rate of 10 mV s^−1^. **b** GCD curves at a current density of 5 A g^−1^. **c** Specific capacitance at various current densities from 1 to 20 A g^−1^. **d** Nyquist plots. The inset is the magnified Nyquist plots at the high-frequency region

The effects of KOH activation and subsequent ammoxidation on the capacitive behaviors of NOAC400, NOAC500, NOAC600, NOAC700, and NOAC800 were investigated by cyclic voltammetry between −1 and 0 V (vs. SCE) at a scan rate of 10 mV s^−1^, galvanostatic charge/discharge at increasing current densities, and electrochemical impedance measurement between 0.01 and 100 kHz as shown in Fig. 4. The distorted segment of CV curves is obvious within the voltage window from −1 to −0.3 V (Fig. 4a), suggesting faradic reactions induced by the heteroatoms (N, O) on the surface and solvated ions penetrating into ultramicropores under strong electrostatic force at high voltage [9, 21]. Besides, the integral area of NOAC400 in the CV curve is the largest among these samples, demonstrating the highest specific capacitance.

GCD tests were carried out in the potential range of −1 to 0 V (vs. SCE) to further compare electrochemical performance of NOAC. Figure 4b represents the GCD curves of the as-prepared samples. The charge/discharge plots exhibit the shape of symmetrical triangle and small IR voltage drops at relatively high current density of 5 A g^−1^, indicating high coulombic efficiency and small equivalent series resistance (ESR). NOAC400 exhibits the longest discharge time compared with the other samples. This phenomenon demonstrates NOAC400 has the highest specific capacitance, which is in accordance with the consequence of CV test. As shown in Fig. 4c, for NOAC400, the specific capacitance is 299 F g^−1^ at 1 A g^−1^, 257 F g^−1^ at 5 A g^−1^, and even reaches 228 F g^−1^ at 20 A g^−1^. In addition, its capacitance retention at current density of 20 A g^−1^ is 76 % of the value at 1 A g^−1^. The superior electrochemical behaviors of NOAC400 are ascribed to its developed porosity, rich N content, and high conductivity (57 S m^−1^). Furthermore, the heteroatom-containing functional groups can promote the wetting properties of material surface and then boost the electrolyte ion transport into the inner micropores.

The evolution of the charge transport property of NOAC400 and other samples was also examined with EIS tests. As depicted in Fig. 4d, the Nyquist plots exhibit an arc in the high-frequency region. The semicircle shown in high frequency is generally caused by the charge transfer resistance of faradic reaction. Especially in the Fig. 4d inset, NOAC400 shows a bigger semicircle radius in comparison with the other samples, which is mainly originated from its highest content of heteroatom. The Warburg region (from 8.3 to 17.8 Hz) exhibits a diagonal line with a slope of 45°, representing typical Warburg impedance. This result indicates porous structure of NOAC400, which can be confirmed by the N_2_ adsorption results. The plot shows a line intersecting with the real axis at a near 90° angle in the low-frequency region, which reflects typical capacitor behavior. The low device internal resistance of 0.44 Ω further proves the high conductivity of the NOAC400, corresponding to its excellent EDLC behavior.

The prominent supercapacitive performance of NOAC400 should be attributed to these reasons as follows: (i) The high surface area could provide a large adsorption interface for electrolyte ions to form EDL, resulting in enhanced specific capacitance; (ii) the introduction of nitrogen-containing groups can not only increase the accessibility of electrolyte ions to make full use of the surface area but also provide considerable pseudocapacitance to enhance the overall capacitance; and (iii) high conductivity induced by N doping guarantees the fast transfer of electric charges, which decreases the device internal resistance and eventually promotes a high-rate property of the supercapacitor [18].

## Conclusions

To take full advantage of PC in clean energy storage, N-doped AC was fabricated by KOH activation and ammonia treatment of PC. The as-prepared carbon possesses high surface area and abundant nitrogen-containing groups, exhibiting excellent supercapacitive performance, such as high specific capacitance and outstanding rate capability. Besides, this treatment strategy can be a promising method in promoting the development and utilization of PC and accelerating its practical application in electrochemical energy storage.
